# Supplementary material for: Effectiveness of Vascular Catheter Removal Versus Retention in Non-ICU Patients with CRBSI or CABSI in Retrospective, Single-Center Study
Source: Microorganisms. 2025 May 7;13(5):1085. doi: 10.3390/microorganisms13051085 (PMC12114613; doi:10.3390/microorganisms13051085)
Supplement: Supplementary file 1 [file microorganisms-13-01085-s001.zip › microorganisms-3547012-supplementary.pdf]

Table S1: Epidemiological characteristics and VAD management of CRBSI and CABSI events, stratified by different catheters

		PICC (N = 60)	CICC (N = 6)	FICC (N = 11)	Midline (N = 87)	All (N = 164)	p-value
Type of infection	CRBSI	39 (65.0)	2 (33.3)	5 (45.5)	26 (29.9)	72 (43.9)	
	CABSI	21 (35.0)	4 (66.7)	6 (54.5)	61 (70.1)	92 (56.1)	
Median lumen number	median [iqr]	1 [1, 2]	1.5 [1, 2]	1 [1, 2]	1 [1, 1]	1 [1, 1]	
Site of insertion	Thigh	10 (16.7)	0 (0.0)	11 (100.0)	57 (65.5)	78 (47.6)	
	Neck	0 (0.0)	5 (83.3)	0 (0.0)	0 (0.0)	5 (3.0)	
	Arm	50 (83.3)	1 (16.7)	0 (0.0)	30 (34.5)	81 (49.4)	
Number of attempts	median [iqr]	1 [1, 1]	1 [1.0, 1.8]	1 [1, 1]	1 [1, 1]	1 [1, 1]	
Microbiological isolates (BCs)							
Polymicrobial infection		12 (20.0)	0 (0.0)	1 (9.1)	12 (13.8)	25 (15.2)	0,459
Staphylococcus aureus		6 (10.0)	0 (0.0)	0 (0.0)	2 (2.3)	8 (4.9)	0,138
CoNS		26 (43.3)	2 (33.3)	6 (54.5)	49 (56.3)	83 (50.6)	0,363
Streptococcus spp.		2 (3.3)	0 (0.0)	0 (0.0)	2 (2.3)	4 (2.4)	0,889
Enterococcus spp.		14 (23.3)	2 (33.3)	4 (36.4)	15 (17.2)	35 (21.3)	0,391
Enterobacteriaceae		11 (18.3)	0 (0.0)	0 (0.0)	11 (12.6)	22 (13.4)	0,269
Pseudomonas aeruginosa		1 (1.7)	0 (0.0)	0 (0.0)	1 (1.1)	2 (1.2)	0,958
Bacillus spp.		1 (1.7)	0 (0.0)	0 (0.0)	2 (2.3)	3 (1.8)	0,933
Candida spp.		9 (15.0)	1 (16.7)	1 (9.1)	11 (12.6)	22 (13.4)	0,939
No microbiological isolate		1 (1.7)	1 (16.7)	1 (9.1)	7 (8.0)	10 (6.1)	0,264
Clinical decision							
VAD removal, no re-positioning		12 (20.0)	0 (0.0)	3 (27.3)	25 (28.7)	40 (24.4)	0,320
VAD removal, re-positioned > 48h after		6 (10.0)	0 (0.0)	0 (0.0)	9 (10.3)	15 (9.1)	0,590
VAD removal, re-positioned < 48 h after		21 (35.0)	5 (83.3)	2 (18.2)	26 (29.9)	54 (32.9)	0,037
No VAD removal + guidewire exchange		1 (1.7)	1 (16.7)	0 (0.0)	0 (0.0)	2 (1.2)	0,004
No VAD removal + lock therapy		1 (1.7)	0 (0.0)	0 (0.0)	0 (0.0)	1 (0.6)	0,627
No VAD removal + lock antibiotic therapy		4 (6.7)	0 (0.0)	1 (9.1)	1 (1.1)	6 (3.7)	0,236
No VAD removal + systemic antibiotic therapy		15 (25.0)	0 (0.0)	5 (45.5)	23 (26.4)	43 (26.2)	0,232
No VAD removal, no antibiotic therapy or lock therapy		0 (0.0)	0 (0.0)	0 (0.0)	3 (3.4)	3 (1.8)	0,439

CRBSI catheter-related bloodstream infection, CABSI catheter-associated bloodstream infection, BCs blood cultures, VAD vascular access device
